# Supplementary material for: Prediction of true Helicobacter pylori-uninfected status using a combination of age, serum antibody and pepsinogen: Logistic regression analysis
Source: PLoS One. 2020 Oct 1;15(10):e0240040. doi: 10.1371/journal.pone.0240040 (PMC7529238; doi:10.1371/journal.pone.0240040)
Supplement: Attachment — Submitted filename: Response to Reviewers.docx [file pone.0240040.s001.docx]

Dear Professor Szecsi,

Thank you very much for reviewing our manuscript and offering valuable advice.

We have addressed your comments with point-by-point responses and revised the manuscript accordingly.

*Prediction of a condition without invasive procedures is always interesting. I myself have also worked with that problem years back however gastroscopy is still the choice.*

**RESPONSE:** I agree with your opinion that prediction without an invasive procedure is important. The paper that you pointed out was added as reference 11.

*A predictive model has rather stick demands, especially with sensitivity if invasive procedure could be avoided. I would like a discussion on negative predictive value in light of the relative low incidence of the disease, even in higher risk areas.*

**RESPONSE:** Thank you for your valuable comment. First, I would like to confirm that “negative predictive value” in your comment might be the proportion of true HP-uninfected subjects with a negative test result who are correctly diagnosed. However, in our study, because we developed the prediction formula for the outcome of true uninfected individuals, test positive = true uninfected individuals, test negative = current infection or spontaneously eradicated cases. Since Reviewer #2 recommended (in comment 4) that we describe the definitions of sensitivity and specificity to avoid confusion, we have included them on p. 6, lines 139–144. We have also added a discussion about positive and negative predictive value. In the present study, the sensitivity, specificity, positive predictive value, and negative predictive value at the optimal cut-off value (0.8135) of the best predicting formula (four-parameter model: age, HP antibody, PGI, and PGII) were 93.2%, 88.5%, 96.5%, and 79.3%, respectively. We found that positive predictive value was sufficient because only 3.5% of test positive subjects with a current infection and those with a spontaneously eradicated infection were misdiagnosed as test positive (true uninfected).

Reviewer #1: This is an interesting study which to discriminate uninfected individuals from the subjects with past or current Hp infection “without endoscopic examination”. These are suggestions to improve the manuscript.

**RESPONSE:** We wish to express our appreciation to the Reviewer for the insightful comments, which have helped us significantly improve the paper.

Major comments:
*1. Main flaw of this study is the discrepancy found between the results and conclusions. Although uninfected subjects were younger in age, showed negative Hp serology test finding, higher serum PG I concentration, and lower PG II concentration, there is no combination of these four significant variables even in Figure 2. Because the authors stick too much to all 6 variables, it was concluded that a combination of 6 noninvasive parameters (sex, age, HP antibody, PGI, PGII and the PGI/II ratio). is useful in predicting true HP uninfected individuals without endoscopic examination. Please do not stick on comparing between the accuracies of 6 variables and 3 variables (HP antibody, PGI and PGI/II ratio).*

**RESPONSE:** We agree with your assessment. We have incorporated this suggestion throughout our paper. We finally determined that the prediction model with the four-parameter combination (age, HP antibody, PGI, and PGII) was the best tool for predicting true HP-uninfected individuals (p. 8, lines 182–183).

*2. Because the “true uninfected individuals” were determined by a negative stool antigen test and the lack of advanced CAG (> closed-type 1) or MG on endoscopic finding, there might be a discrepancy with the results derived by using 6 noninvasive parameters (sex, age, HP antibody, PGI, PGII and the PGI/II ratio). For clarification, the cutoff values of age, serum PG I level, and PG II level should be shown with their accuracies.*

**RESPONSE:** Thank you for providing this insight. Since the present study aimed to estimate the true uninfected individuals, I think it is inevitable that there is a discrepancy between the four noninvasive parameters and negative stool antigen test and endoscopic findings. We have added new Table 3, which shows the cut-off value of each parameter and their combinations.

*3. For better clinical application of the prediction formula, 467 subjects with past eradication should be tested as well.*

**RESPONSE:** You have raised an important point; however, we believe that 467 subjects who had undergone eradication therapy would be outside the scope of our paper because this prediction model was made to substitute for the ABC classification. In addition, this population is heterogeneous, which leads to misclassification.

The present study follows exclusion criteria of the ABC classification. In Japan, when patients visit healthcare centers, we ask them in advance whether they have a history of *H. pylori* eradication therapy. If not, we apply the ABC classification to them. This is because, without excluding individuals who have undergone eradication therapy, some such individuals might be misclassified into Group A (true uninfected group), since data (HP antibody and PGs) from these individuals are similar to those of true uninfected individuals. However, we analyzed the subjects including those who had undergone eradication therapy based on your suggestion as follows.

A flowchart of the study including post-*H. pylori*-eradication-therapy individuals is shown in Fig 3 (only for rebuttal letter). We excluded 78 patients who did not undergo endoscopy, 4 patients who did not undergo SAT, 1 patient who did not undergo PG testing, 7 patients who had a history of gastrectomy, and 46 patients who were taking proton pump inhibitors. Finally, 331 patients who had undergone eradication therapy were added to the 684 patients.

Fig 3 (only for rebuttal letter). Flowchart of the study (including post-*H. pylori*-eradication-therapy individuals)

We next performed a logistic regression analysis with the combination of age, sex, HP antibody, and PGs. Factors significantly associated with true uninfected status were sex, age, HP antibody, PGI, and PGII (Table 5: only for rebuttal letter).

|  | Odds ratio | 95% CI | | *p* value |  |
| --- | --- | --- | --- | --- | --- |
| **Sex** | 0.5548 | 0.3950 | 0.7793 | <0.001 | ** |
| **Age** | 0.9056 | 0.8905 | 0.9209 | <0.001 | ** |
| **HP antibody** | 0.6572 | 0.5987 | 0.7215 | <0.001 | ** |
| **PGI** | 1.0348 | 1.0194 | 1.0504 | <0.001 | ** |
| **PGII** | 0.9036 | 0.8225 | 0.9927 | 0.0347 | * |
| PGI/II ratio | 0.9597 | 0.8880 | 1.0372 | 0.2994 |  |

Table 5 (only for rebuttal letter). Variables associated with true uninfected status in logistic regression analysis (N=1015, including post-eradication-therapy individuals). **p* <0.05, ***p* <0.001.

AUC (95% CI) was 0.8835 (0.863–0.904) upon the application of sex, age, HP antibody, PGI, and PGII. At the optimal cut-off value from the ROC curve, the sensitivity and specificity were 82.2% and 79.3%, respectively (Fig 4, only for rebuttal letter).

Fig 4 (only for rebuttal letter). AUC for true uninfected individuals with sex, age, HP antibody, PGI, and PGII.

The diagnostic accuracy (AUC, sensitivity, and specificity) of Fig 4 was inferior to that of Fig 2. The results suggested that, the greater the number of post-eradication-therapy individuals included among the subjects, the greater the number of misclassified post-eradication-therapy individuals in the true uninfected group. Therefore, we should exclude as many patients who have undergone eradication therapy as possible in advance before applying the prediction model.

Again, because this prediction model follows the exclusion criteria of the ABC classification, and was developed for patients without a history of eradication therapy, researchers should ensure the exclusion of patients with a history of eradication therapy before applying the model.

Minor comments:
*1. Rewrite the Methods and Results under the subheading, and please do not insert the tables and figures between the main body. Put them at the end of the manuscript.*

**RESPONSE:** Thank you for your comment. We have rewritten the Methods and Results with subheadings and placed the tables and figures at the end of the manuscript.

*2. The title looks less scientific because of “other factors”.*

**RESPONSE:** We agree with your assessment. We have changed the title to “Prediction of true *Helicobacter pylori*-uninfected status using a combination of age, serum antibody and pepsinogen: Logistic regression analysis.”

Reviewer #2: The authors developed accurate HP prediction model. The prediction model had higher sensitivity and specificity than conventional model. However, The authors did not explain the meaning of HP prediction. Accurate HP prediction model cause effective screening. The authors must discuss the usefulness of the study.

**RESPONSE:** Thank you very much for providing important insights. We are grateful for the time and energy you expended on our behalf.

*1. The purpose of ABCD classification is not only prediction of HP infection status, but also evaluation of gastric cancer risk. Risk stratification by ABCD classification might enable personalized prevention of gastric cancer. The new HP status prediction might lead better gastric cancer screening, however, nothing was described concerning gastric cancer prevention.*

**RESPONSE:** We agree with you and have incorporated this suggestion on p. 8, lines 187–190.

*2. Age was one of the significant variable to predict HP infection in the study. However, some evidences suggested birth cohort (birth year) rather than age was truly associated with HP infection. I am interested in the model including birth cohort as a predictor.*

**RESPONSE:** Thank you for the thoughtful feedback. We have read the papers about *H. pylori* prevalence and a birth cohort pattern **(**Miyamoto R, et al. Rapidly decreasing prevalence of Helicobacter pylori among Japanese children and adolescents: J Infect Chemother. 2019:25(7): 526-530) **(Wang C, et al. Changing trends in the prevalence of H. pylori infection in Japan (1908-2003): a systematic review and meta-regression analysis of 170,752 individuals: Sci Rep. 2017;7(1): 1-12).**

We suppose that the diagnostic accuracy would not change much irrespective of whether we chose age or birth year as a predictor in the present study because our study is on a cohort from a short time period (2017–2019).

We would like to explain this with some examples. In the present study, the HP infection rates of a 58 y/o patient in 2017 (birth year 1959, prevalence 50.5% according to Wang’s report) and a 58 y/o patient in 2019 (birth year 1961, prevalence 47.7%) were almost the same. Therefore, the results of our study would not change irrespective of whether we choose age or birth year as an explanatory variable. However, when studying a long-term cohort, we agree with you that it would be more accurate to use birth year instead of age as a variable. For example, assuming that the study was conducted from 2009 to 2019, there would be a 10.9% gap between the HP infection prevalence of a 58 y/o patient in 2009 (birth year 1951, prevalence 58.6%) and a 58 y/o patient in 2019 (birth year 1961, prevalence 47.7%).

For this reason, birth year might be a better predictor of HP infection. However, if we were to use birth year as a predictor, we should reassess the prediction formula each time because the HP infection prevalence of the target population is continuously changing. For instance, the prediction model that was established from the target population from 1908 to 1958 (yellow square in Fig 5) cannot be applied to the target population from 1940 to 1990 (green square in Fig 5) because the correlations between birth year and HP infection prevalence differ. In addition, Wang et al. reported that the HP infection prevalence curve differs among countries.

Since we cannot tell the prevalence of H. pylori infection in subsequent generations and prevalence differs from place to place, **we believe that the prediction model including age or birth year should be assessed in each studied time period and geographical region.** We have added a discussion about this on p. 9, lines 208–214.

Fig 5 (only for rebuttal letter). Predicted prevalence of *H. pylori* infection in Japanese population by birth year from 1908 to 2003 from the study by Wang et al.

*3. Sex was included in the 6-parameter prediction formula, although it did not have significant impact on HP infection status. Did it improve the discriminatory ability of the model?*

**RESPONSE:** Thank you for providing this insight. We have added new Table 3, which shows the AUC (95% CI), cut-off value, sensitivity, and specificity of each variable and their combinations. Even when adding sex to the combination model of age, HP antibody, PGI, and PGII or age, HP antibody, PGI, PGII, and PGI/II ratio, the diagnostic accuracy did not change [AUC 0.944 vs. AUC 0.943, *p*=0.700 (Fig 2)]. In conclusion, sex did not improve the discriminatory ability of the model, so we removed it.

*4. Describe a definition of sensitivity and specificity in the study. In usual case, sensitivity means a proportion of test positives among diseased subjects and specificity means a proportion of test negatives among healthy subjects. Although no definition of sensitivity and specificity in the study, I guessed a proportion of uninfected defined by test (true uninfected) among all uninfected subjects was sensitivity. Thus, the definition of sensitivity and specificity were opposite to the usual ones. It would confuse readers to assess the results.*

**RESPONSE:** Thank you for your suggestion. We have added definitions of sensitivity and specificity (p. 6, lines 139–144).

*5. The study design aimed to specify true uninfected population. Why the authors specify uninfected population by the prediction model? Some intervention would be necessary for HP infected group to prevent gastric cancer, while uninfected people do not need any help. Therefore, it is natural to find HP infected (current HP infection + spontaneously eradicated) by the prediction model.*

**RESPONSE:** Thank you for your suggestion. We specified the uninfected population by the prediction model because these individuals have a low risk of gastric cancer and do not need to undergo endoscopy, which is an invasive procedure. By this prediction model, we are also able to specify HP-infected cases (current HP infection + spontaneously eradicated cases). Since the optimal cut-off value for true uninfected individuals is 0.8135, the patients with P <0.8135 can be defined as HP-infected (current infection or spontaneously eradicated cases). We have responded to this comment by adding the definition of P <0.8135 (p. 7, lines 155–158).

*6. Suppose the prediction model is used for gastric cancer prevention, high risk population (HP infected population) should be detected for effective screenings. Examination with high sensitivity was relevant for screening despite of relatively less specificity, because of less false negatives. As for gastric cancer screening, minimum number of cases who were HP infected but judged as HP uninfected by examinations, should be required. Were the cut-off value of 91.3% sensitivity and 89.7% specificity appropriate?*

**RESPONSE:** You have raised an important point. We compared the diagnostic accuracy between cut-off values of 0.8135 and 0.90 because 0.90 was expected to have higher specificity (fewer cases with HP infection but judged as HP-uninfected by the prediction formula). The sensitivity and specificity results for a cut-off value of 0.8135 were 93.2% and 88.5%, while those for a cut-off value of 0.90 were 79.7% and 91.7%, respectively. Although the specificity of the cut-off value of 0.90 was higher than that of 0.8135, the sensitivity of the former was markedly lower than that of the latter. Therefore, we concluded that 0.8135 was the most appropriate cut-off value. We have incorporated your suggestion by adding Table 4 and text on p. 7, lines 165–174.

Again, thank you for giving us the opportunity to strengthen our manuscript with your valuable comments and queries. We have worked hard to incorporate your feedback and trust that the revised manuscript is suitable for publication.

Sincerely

Takako Takayama

Hideo Suzuki
